# Rumen DNA virome in beef cattle reveals an unexplored diverse community with potential links to carcass traits

**DOI:** 10.1093/ismeco/ycaf021

**Published:** 2025-02-05

**Authors:** Yoshiaki Sato

**Affiliations:** Department of Agrobiology and Bioresources, School of Agriculture, Utsunomiya University, 350 Minemachi, Utsunomiya, Tochigi 321-8505, Japan

**Keywords:** carcass traits, cattle, microbiome, rumen, virome

## Abstract

Rumen deoxyribonucleic acid viruses that infect and replicate within bacteria and archaea are key modulators of the prokaryotic community. These viruses influence prokaryotic community abundance, composition, and function impacting host productivity and methane production. In this study, viral genomes were assembled from the rumen of 37 Japanese Black cattle using virus-like particle metagenome sequencing, providing insights into viral diversity, functional potential, and virus–host interactions. The relationship between the rumen deoxyribonucleic acid virome and carcass traits, particularly carcass weight and marbling, was also investigated. A total of 22 942 viral operational taxonomic units of medium-quality or higher (≥5 kb length and ≥ 50% completeness), referred to as Japanese Black Rumen Viral genomes, were reconstructed. Among these, 5973 putative novel genera were identified, significantly expanding the catalog of rumen viral genomes. Hosts were predicted for 2364 viral operational taxonomic units, including carbohydrate-degrading bacteria and methanogens. Additionally, 27 auxiliary metabolic genes were categorized as glycosyl hydrolases which are responsible for the degradation of cellulose, hemicellulose, and oligosaccharides, suggesting that rumen viruses may enhance the breakdown of complex carbohydrates during infection. Furthermore, the rumen virome differed considerably between high vs low carcass weight cattle and high vs low marbling cattle. Viruses associated with *Methanobrevibacter* were linked to higher carcass weight. This database and the insights from this study provide primary information for the development and improvement of beef production.

## Introduction

Ruminants have a complex rumen microbial community; therefore, they can degrade fibers that are difficult to digest. The rumen microbial ecosystem comprises bacteria, archaea, fungi, protozoa, and viruses [1]. Bacteria primarily produce volatile fatty acids, which serve as an energy source for the host, and microbial nitrogen, essential for the growth and production of animal proteins, such as meat and milk. Archaea are responsible for methane production during anaerobic fermentation. Previous studies have extensively studied the correlation between host traits and rumen microbiome given the advances in next-generation sequencing technologies. For instance, the rumen prokaryotic community has been linked to host breeds [2], carcass traits of beef cattle [3–5], milk production [6, 7], and methane yield [8, 9].

In the rumen, viruses, especially tailed viruses, are highly abundant, with previous studies reporting 2 × 10^7^ to 1.6 × 10^10^ particles/mL of ruminal fluid [10–12], and many of these are viruses that infect and replicate within bacteria and archaea, representing a significant component of the rumen microbiome ecosystem. These viruses are expected to play crucial roles in shaping microbial communities through cell lysis and horizontal gene transfer [13]. Consequently, rumen viruses are considered key modulators of the prokaryotic community, potentially influencing its abundance, composition, and function.

Our understanding of the diversity of the rumen deoxyribonucleic acid (DNA) virome has advanced due to the development of large reference genome databases, such as the rumen virome database (RVD) [14] and rumen virus genome (RVG) [15], which were constructed using bulk metagenomes and virus-like particle (VLP) metagenome sequencing, respectively. These studies demonstrated that the rumen virome is highly diverse, with viruses correlating with prokaryotes involved in carbohydrate degradation and methane production. Moreover, some viruses in the rumen harbor virus-encoded auxiliary metabolic genes (AMGs), which are expressed during infection to enhance host metabolism and viral production. For example, certain AMGs encode glycoside hydrolases (GHs) associated with lignocellulose degradation, such as cellulases and xylanases [14–16]. Additionally, some rumen viruses contain AMGs involved in methane metabolism, potentially modulating host methane production [17]. Given the central role of prokaryotes in carbon metabolism within the rumen ecosystem, viruses may play a critical role in carbon cycling and indirectly influence host productivity and methane production; however, these roles remain largely overlooked.

The rumen virome is influenced by the host’s condition and environmental factors. A previous study revealed that host breeds can shape the virome, as evidenced by comparisons between the rumen viromes of Japanese Black cattle and crossbreeds (Japanese Black sires × Holstein dams) [15]. Furthermore, the rumen virome is responsive to dietary shifts [16, 18], and has been linked to feed efficiency, lactation performance, weight gain, and methane emissions [18]. In beef cattle, the composition and function of the rumen prokaryotic community are associated with marbling and carcass weight (CW) [3–5]. Notably, prokaryotes such as *Prevotella* and *Methanobrevibacter* involved in carbon [19–21] and methane metabolisms [20, 22, 23] play a significant role in these carcass traits [3]. Since the rumen viruses can affect the rumen prokaryotic community [14–16], it is plausible that viruses infecting prokaryotes related to carbon and methane metabolism may also influence carcass traits. However, this relationship remains unclear. To address this knowledge gap, this study first assembled DNA viral genomes from the rumen of 37 Japanese Black cattle using VLP sequencing to gain insights into their diversity, functional potential, and virus–host interactions. Additionally, the study also investigated the relationship between the rumen DNA virome and carcass traits, particularly CW and marbling, in Japanese Black cattle. This study has resulted in a valuable database of novel rumen viral genomes and new insights into how rumen viruses influence carcass traits.

## Materials and methods

### Ruminal fluid sampling and sample data

The 37 of 55 rumen liquids collected from a commercial slaughterhouse in a previous study [3] were used for virome analysis. According to the CW and beef marbling standard (BMS), which is used to evaluate beef marbling in Japan, the Japanese Black cattle were classified as having high CW (HW; *n* = 12, CW = 617.0 ± 39.59 kg), low CW (LW; *n* = 12, CW = 488.4 ± 20.46 kg), high marbling (HM; n = 13, BMS scores ≥11), and low marbling (LM; *n* = 12, BMS scores ≤6) [3]. Ethical approval was not required for the current study, as the samples were obtained from animals killed for commercial purposes unrelated to this study.

### Virus-like particles enrichment, deoxyribonucleic acid extraction, and metagenomic sequencing

VLPs enrichment and DNA extraction were performed using a previously reported method [24] with modifications for the ruminal samples. Briefly, 3 mL rumen samples were centrifuged twice at 6000 × *g* for 30 min at 4°C to remove large debris. The supernatants were filtered through a 0.45-μm followed by a 0.22-μm polyvinylidene difluoride filter (Millipore, Burlington, MA, USA). The filtrate was mixed with an equal volume of 20% polyethylene glycol solution (PEG-6000-2.5 M NaCl) and stored at 4°C overnight. The fluid was centrifuged at 20380 × *g* for 45 min at 4°C, and the supernatant was discarded. The VLP pellet was suspended in 215 μL of 0.02 μm-filtered saline-magnesium buffer containing 5 mg/mL lysozyme and incubated for 60 min at 37°C to degrade the remaining bacterial cell membranes. The lysate was treated with 12 U DNase (Nippon Gene), 5 U TURBO DNase (Thermo Fisher Scientific), 5 U Baseline-ZERO DNase (Epicentre), 25 U Benzonase (Sigma Aldrich), and RNase (25 g/sample; Nippon Gene) for 1 h at 37°C. Enzyme activity was ceased by adding ethylenediaminetetraacetic acid (final concentration of 20 mM) and incubating at 70°C for 15 min. Sodium dodecyl sulfate (final concentration of 1%) and proteinase K (final concentration of 0.5 mg/mL; TaKaRa Bio) were added. The mixture was incubated at 55°C for 20 min, followed by incubation at 70°C for 5 min. Genomic DNA was extracted using a combination of phenol: chloroform: isoamyl alcohol (25:24:1) and chloroform: isoamyl alcohol (24:1) protocols. DNA was precipitated by the addition of 3 M sodium acetate, isopropanol, and Dr. GenTLE precipitation carrier (Takara Bio). After washing with 70% ethanol, the dried DNA pellet was suspended in 0.02 μm-filtered MilliQ water. Metagenomic libraries were prepared using an Illumina DNA Prep and Tagmentation Kit (Illumina, San Diego, CA, USA). Libraries were sequenced using the Illumina HiSeq X Ten (2 × 150-bp) or NovaSeq X Plus (2 × 150-bp).

### Determination of viral operational taxonomic units

Low-quality reads and adapters in the raw data were filtered using Trimmomatic v0.39 [25]. To remove host sequences, the trimmed reads were aligned against the reference bovine genome ARS-UCD1.2/bosTau9 (GCF_002263795) using BWA-MEM [26], collecting the unmapped reads. Subsequently, contigs were assembled with SPAdes v3.15.3 in metagenomic mode [27, 28]. Contigs longer than 5 kb were collected for further analysis. The resulting contigs from each sample were pooled together and clustered at 95% identity and 85% coverage using CD-HIT v4.8.1 [29] to viral operational taxonomic units (vOTUs), representing species-level viral clusters (VCs) [30]. Viruses were predicted from non-redundant contigs using VirSorter2 v2.2.2 [31]. Subsequently, the completeness evaluation and host fraction removal of vOTUs were performed using CheckV v0.8.1 [32]. The vOTUs that were attributed to “complete”, “high-quality” (≥90% completeness), or “medium-quality” (50%–90% completeness) were used for downstream analyses (referred to as Japanese Black Rumen Viral genomes [JBRV]).

### Functional annotation of viral operational taxonomic units

Protein prediction of vOTUs was performed using Prodigal v2.6.3 using the “-p meta” option [33]. The predicted proteins were annotated against PHROGS v4 [34] using MMseqs2 v13.45111 [35], and the top hit annotations and categories with the lowest e-values (< 0.001) were chosen. Lifestyle classification was performed using PhaTYP [36].

### Identification of auxiliary metabolic genes

To be conservative, high confidence vOTUs, which has three or more geNomad virus hallmark markers [37], were used for identification of viral AMGs. DRAM-v [38] and VIBRANT [39] were used to recover putative AMGs from vOTUs. Before AMG analysis using DRAM-v [38], vOTUs were reprocessed using VirSorter2 with the “—prep-for-dramv” option to produce the “affi-contigs. tab” files and then annotated using the DRAM-v [38]. For the DRAM-v output, genes with an auxiliary score of <4 and an M flag were obtained, while those with transposon regions (T flag) were excluded. Genes with an auxiliary score of 1 were classified as *bona fide* AMGs, while genes with auxiliary scores of 2 or 3, as well as VIBRANT outputs, were manually curated to identify *bona fide* AMGs, which were characterized by being flanked by geNomad virus hallmark markers. Genome linear maps were visualized using CGView [40] on the Proksee web server [41].

### Taxonomic classification and viral protein cluster determination

Rumen viral genomes with more than medium-quality in RVD and RVG were used for comparison with JBRV vOTUs. Clustering of JBRV with RVD, RVG, and RefSeq viral genomes (−db ProkaryoticViralRefSeq201-Merged database) was performed using vConTACT2 v.0.9.19 [42]. JBRV vOTUs that were classified into ambiguous clusters (i.e. outliers and overlap) were referred to as “single vOTU”, and each “single vOTU” was considered as one VC. Taxonomic annotations of JBRV vOTUs were conducted using geNomad [37].

### Prediction of virus–host relationships

This study constructed a reference host database including rumen prokaryotic genomes from the Hungate1000 project [43] and metagenome-assembled genomes (MAGs) from the gastrointestinal microbiome of ruminants reported in previous studies [3, 44–47], which includes prokaryotic genomes in the rumen of the cattle used in this study. Furthermore, archaeal genomes in RefSeq were used to extend the archaeal host database for host prediction. The prokaryotic genomes were taxonomically annotated using GTDB-tk v.2.1.1 [48]. Clustered Regularly Interspaced Short Palindromic Repeats (CRISPR) spacer sequences with evidence levels of 3 or 4 were extracted using CRISPRCasFinder v.4.2.20 [49]. The predicted CRISPR spacers were aligned against JBRV vOTUs using blastn-short from BLAST+ v2.9.0 [50], and matches with ≥95% identity of the spacer length and < 2 mismatch were retained.

### Japanese Black cattle rumen viral community

The filtered reads of VLPs sequencing from 37 samples were mapped to JBRV vOTUs using BamM “make” v1.7.3 (https://github.com/ecogenomics/BamM), and the reads that aligned over ≥80% of their length at ≥95% nucleic acid identity were screened using the BamM “filter.” The read counts were determined using CoverM v.0.4.0 (https://github.com/wwood/CoverM). Breadth coverage was determined using BEDtools “genomecov” v2.30.0 [51] and read counts of vOTUs covered at <80% were set to zero. Reads per kilobase per million mapped reads (RPKM) values were calculated. To calculate the abundance of each VCs, the RPKM values of the same VCs were summed. The relative abundances of vOTUs and VCs were calculated using RPKM. vOTUs and VCs that were present in >50% of the samples were considered as “core” vOTUs and VCs. Correlation analyses between core VCs and carcass traits were performed using Spearman’s correlation in R. Differences in the relative abundance of core VCs correlated with carcass traits were tested using the Mann–Whitney U test, and the p-values were adjusted using Benjamin-Hochberg’s method (q-value).

The vOTUs and VCs accumulation curves were calculated using the specaccum function with 1000 permutations in the “vegan” package in R [52]. The number of vOTUs and Shannon diversity index [53] were deduced using the “vegan” package [52]. Non-metric dimensional scaling ordinations (NMDS) based on Bray–Curtis dissimilarity were performed using the “vegan” package [52]. Statistically significant differences between carcass traits were tested using Mann–Whitney U test and permutational multivariate analysis of variance (PERMANOVA) with 9999 permutations for alpha and beta diversities, respectively. Differences were considered statistically significant at *P* < .05 or q < 0.1.

## Results and discussion

### Construction of JBRV

From VLPs-based metagenomic sequencing on rumen liquids of 37 Japanese Black cattle, 167 227 non-redundant contigs >5 kb in length were constructed by assembly and clustering at 95% identity and 85% coverage. After viral identification using Virsorter2 [31], 149 817 contigs (89.6%) were identified as potential viral candidates. This suggests that the VLPs purification method used in this study is adaptable to rumen samples, similar to the procedure using CsCl density gradient ultracentrifugation reported in a previous study [15].

**Figure 1:**
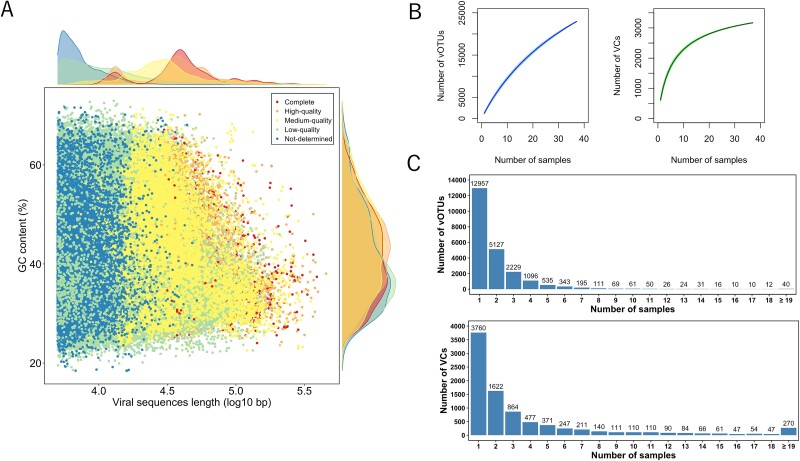
Overview of rumen viral genomes in JBRV. (A) Scatter plot illustrating the distribution by genome length and GC content of vOTUs classified using CheckV. (B) Accumulation curves for vOTUs and VCs from the JBRV genomes database. (C) Distribution of vOTUs and VCs across hosts.

Furthermore, 22 942 vOTUs were classified as medium-quality or higher using CheckV [32], including 2895 complete, 6061 high-quality, and 13 986 medium-quality vOTUs (Fig. 1A; Supplementary Table 1). The quality of these vOTUs in the rumen may be underestimated because CheckV estimates completeness by comparing sequences with a database of complete viral genomes, mostly derived from other ecosystems [14]. There are two large rumen viral genome databases (RVD and RVG). RVD includes 397 180 rumen viral genomes derived from sequences of bulk metagenomes [14], whereas RVG, which is the only database from VLPs-sequencing, comprises 8232 viral genomes [15]. These databases illustrate the large diversity of the rumen viral community. However, significant parts of the rumen virome may be uncharacterized. Given the distinct discrepancies between the viral genomes reconstructed by VLPs and bulk metagenomics [54], reconstructing more viral genomes from VLPs-sequencing is essential to provide a complementary view of the rumen virome. JBRV has expanded the viral genomes from VLPs-sequencing approximately 3-fold compared to those from RVG and will be a valuable resource for future studies investigating the role of the rumen virome.

The JBRV length was in the range of 5016–454 592 bp, with an average length of 41 380 bp. The 197 vOTUs with a length of >200 kb conformed to the definition of jumbo viruses [55]. Of the JBRV, 9839 vOTUs (42.9%) were classified as temperate viruses, consistent with the findings of a previous study, which reported that the rumen virome has a comparatively high prevalence of lysogeny [15]. According to the accumulation curve, although the VC accumulation curve gradually transitioned to a plateau with an increased sample number, the vOTUs accumulation curve did not reach a plateau (Fig. 1B). Additionally, the mapping rate against JBRV was only 36.4 ± 7.10% (mean ± standard deviation), suggesting that the JBRV had not fully represented the rumen virome due to the highly individual-specific signature of the rumen virome [14, 15]. Furthermore, 12 957 vOTUs and 3760 VCs were present in one sample, whereas only 40 core vOTUs and 270 VCs were detected (Fig. 1C). An increasing number of samples is required to comprehensively capture rumen virome diversity. After taxonomic analysis, most vOTUs in JBRV (22 533 vOTUs, 98.2%) were classified as Caudoviricetes (Supplementary Table 1), suggesting that rumen is dominated by the tailed DNA phages, consistent with findings from previous studies [14–16, 56, 57].

### Viral cluster and novelty of JBRV

**Figure 2:**
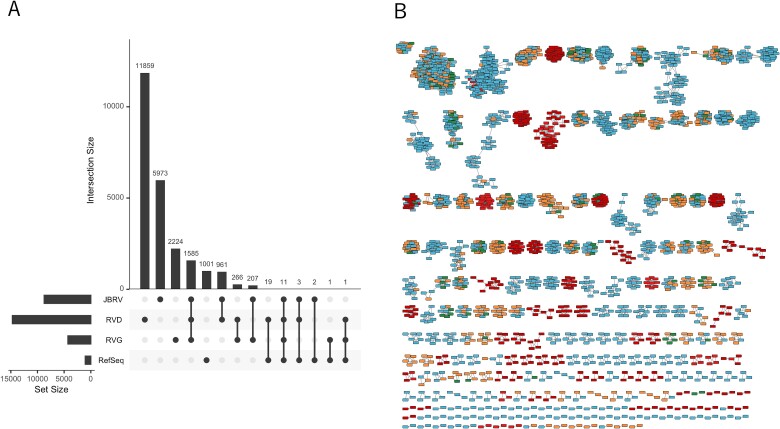
Comparison of the JBRV with RefSeq viral genomes and other ruminal viruses. (A) Upset plot showing the shared VCs among the JBRV, RVD, RVG, and RefSeq databases. VCs included single vOTUs. (B) Gene-sharing networks of viral genomes. The nodes colored orange, light blue, green, and red were viruses in JBRV, RVD, RVG, and RefSeq, respectively.

This study generated “VCs” based on shared protein similarities from JBRV, RVD, RVG, and RefSeq viral genomes using vConTACT2 [42]. The VCs indicate that the viruses may belong to the same viral genera. JBRV vOTUs were clustered into 8742 VCs, including 5573 single vOTUs. Among them, 5973 VCs were unique to JBRV, suggesting that the unique VCs in JBRV represent novel putative genera (Fig. 2A; Supplementary Table 1). Contrastingly, 11 859 VCs were unique to RVD, probably due to the different sequencing approaches used: VLP sequencing for JBRV and bulk sequencing for RVD. Furthermore, 2753 VCs were shared between JBRV and RVD, RVG, or both, whereas only 16 VCs were shared between JBRV and the RefSeq database (Fig. 2A). Additionally, the gene-sharing network revealed that rumen vOTUs were interconnected and distinct from RefSeq viral genomes (Fig. 2B), suggesting that most VCs are rumen-specific and have not been described, consistent with findings from a previous study [15].

### Host-virus interaction

**Figure 3:**
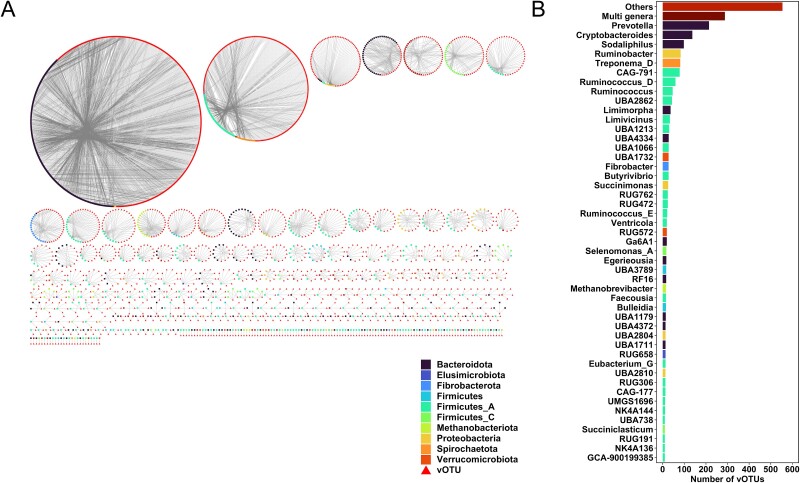
Predicted host-virus interactions in the Japanese Black rumen viral genomes. (A) Network analysis of the host and vOTUs. (B) Predicted hosts of vOTUs at the genus level.

Prokaryotic hosts of JBRV vOTUs were predicted using 200 429 CRISPR spacers from prokaryotic genomes or MAGs from ruminant gastrointestinal samples [3, 43–47] and the RefSeq archaeal database. Hosts of 2364 JBRV vOTUs (10.3%) were predicted, resulting in 4612 virus–host linkages with 1204 prokaryotic genomes (Supplementary Table 2), consistent with the findings from a previous study that predicted putative prokaryotic hosts for a small fraction of vOTUs by CRISPR spacers match [15, 56, 57]. Considering that rumen viruses specifically infect rumen prokaryotes [15], the rumen prokaryote MAGs database used in this study should be extended to predict more virus–host linkages. Among the 1204 prokaryotic host genomes, 815 were associated with more than two vOTUs. These attacks by multiple vOTUs may make it difficult to achieve tolerance. Most vOTUs had prokaryotic hosts belonging to only one phylum (Fig. 3A; Supplementary Table 1). Additionally, 2076 vOTUs were related to only one genus, whereas 288 vOTUs were associated with multiple genera (Fig. 3B), indicating that most vOTUs in the rumen are specialists, while some vOTUs have a broad host range at the genus level.

Putative prokaryotic hosts of JBRV vOTUs were distributed across three archaeal and 18 bacterial phyla (Supplementary Table 1). Among the specialists associated with a single genus, the most frequently predicted hosts were Firmicutes_A (870 vOTUs), followed by Bacteroidota (800 vOTUs) and Proteobacteria (160 vOTUs), which are the dominant phyla in the rumen of the Japanese Black cattle used in this study [3]. At the genus level, *Prevotella* was the most common putative host (214 vOTUs), followed by *Cryptobacteroides* (137 vOTUs) and *Sodaliphilus* (98 vOTUs), all belonging to Bacteroidota (Fig. 3B). These genera are primarily associated with carbohydrate degradation in the rumen. Previous studies [3, 47] have identified rumen MAGs belonging to these genera, which contain numerous polysaccharide utilization loci, operons, or regulons of genes that encode the protein machinery for binding, degrading, transporting, and utilizing polysaccharide substrates [58]. Notably, *Prevotella* vOTU (vOTU9478) was identified as a core vOTU, and many viruses associated with *Prevotella* have been found in the rumen in the previous studies [14, 15, 57], suggesting an important role in carbohydrate metabolisms in the rumen.

For archaea, only 29 specialist vOTUs were associated with the Methanobacteriota (27 vOTUs), Halobacteriota (one vOTU), and Thermoproteota (one vOTU) phyla (Supplementary Table 1 and 2). Most hosts within Methanobacteriota were *Methanobrevibacter*, *Methanobrevibacter_A*, and *Methanobrevibacter_B*, which are assigned to the genus *Methanobrevibacter* according to NCBI taxonomy. The genus is the dominant methanogen in the rumen [22]. Notably, vOTU5553, belonging to VC2781, the host of which was *Methanobrevibacter*, was a core vOTUs, suggesting that it is possible to control the abundance of methanogens in the rumen.

While this study provides valuable insights into virus–host interactions in the rumen through CRISPR spacer matching, the method relies on computational analyses and lacks direct experimental confirmation. Further studies should focus on isolating rumen viruses to obtain direct evidence of host-virus interactions, enabling a more robust understanding of host specificity, infection mechanisms, and the ecological roles of viruses in the rumen microbiome.

### Functional characteristics and identification of auxiliary metabolic genes

To investigate the functional characteristics of the JBRV vOTUs, 1 337 196 predicted proteins were annotated against the PHROGS database. Among them, only 144 466 proteins (10.8% of total proteins) were assigned to the PHROGS database. Notably, 43 210 proteins were classified as “unknown function” (29.9% of annotated proteins), suggesting that most of the proteins in the rumen virome were unidentified (Supplementary Table 3). The most functionally annotated proteins were associated with “DNA, RNA, and nucleotide metabolism” (27.7% of annotated proteins), followed by categories related to “head and packaging” (11.9% of annotated proteins) and “tail” (11.0% of annotated proteins), which are involved in virion production and host lysis, respectively. Six proteins were annotated as pseudomurein endoisopeptidase, an enzyme involved in the lysis of methanogen cell walls. These proteins were found in vOTUs belonging to VC2778, VC4754, VC4755, and VC5218, suggesting that these VCs may be associated with methanogens. Indeed, except for VC5218, these VCs included vOTUs associated with Methanobacteriota (Supplementary Table 1).

Furthermore, 6283 proteins were predicted to be AMGs (Supplementary Table 4), indicating an expansion of AMGs in the rumen compared with the findings reported by recent studies [14, 15]. In this study, two tools, namely DRAMv [38] and VIBRANT [39], were used to predict the AMGs; only 1039 AMGs were identified as overlapping between the two tools. This highlights the limited concordance between the outputs of these tools and underscores the importance of manual curation to eliminate false positives. In many previous studies, AMG analyses using these tools were performed without manual curation [59, 60]. In contrast, we conducted AMG analysis using high confidence vOTUs and applied stringent manual curation criteria, requiring AMGs to be flanked by geNomad virus hallmark markers, ensuring a more conservative approach. However, as suggested in a previous study [14], even stricter criteria might be necessary to confidently exclude false positive AMGs and enhance the robustness of AMG identification.

**Figure 4:**
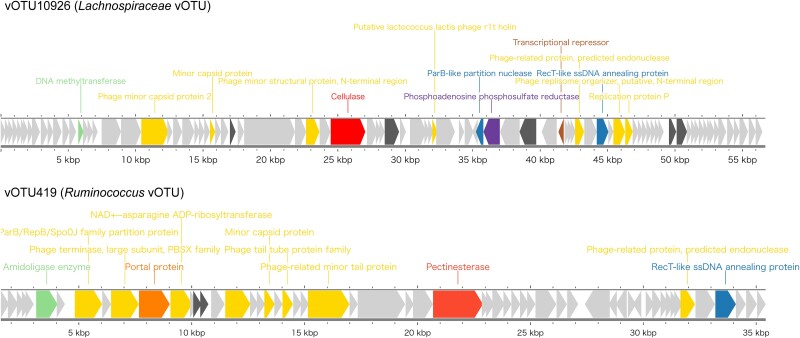
The linear genome map of representative vOTUs with AMGs associated with carbohydrate metabolism. The color of each gene indicates the functional categories: AMG (red), DNA, RNA and nucleotide metabolism (blue), head and packaging (orange), transcription regulation (brown), moron, auxiliary metabolic gene and host takeover (purple), other (green), unknown function (black), hypothetical proteins (gray), and geNomad virus marker (gold).

Most AMGs were annotated as dUTP pyrophosphatase (K01520; 903 AMGs), followed by ribonucleoside-triphosphate reductase (K21636; 672 AMGs), DNA (cytosine-5)-methyltransferase 1 (K00558; 472 AMGs), and phosphoadenosine phosphosulfate reductase (K00390; 427 AMGs), consistent with the findings of a previous rumen virome study [14, 15]. To further explore AMGs associated with carbohydrate metabolism in the rumen, 580 AMGs annotated as carbohydrate-active enzymes were identified across 557 vOTUs. Among these, 248 AMGs were categorized as GHs. Specifically, 27 AMGs were involved in GH2, GH5, GH16, GH39, and GH43, which are responsible for the degradation of cellulose, hemicellulose, and oligosaccharides. Additionally, 3 and 1 AMGs were annotated as pectinesterase (K01051) and polygalacturonase (K01184), respectively, which are associated with pectin degradation. These AMGs, when expressed by vOTUs during or after infection, may enhance the host’s ability to break down complex carbohydrates. Notably, vOTU10926 associated with Lachnospiraceae, known for their potential to degrade cellulose [3], contained GH5 (cellulase) AMGs (Fig. 4). This suggests that the vOTU could contribute to cellulose degradation in the rumen. Pectinesterase AMGs were found in vOTU419 associated with *Ruminococcus* (Fig. 4), a key cellulose utilizer in the rumen, which can degrade pectin to allow access to cellulose. These pectin-degrading enzymes may help break down pectin, facilitating access to cellulose and further promoting fiber digestion [61].

### Viral communities and relationships with carcass traits in Japanese Black cattle

**Figure 5:**
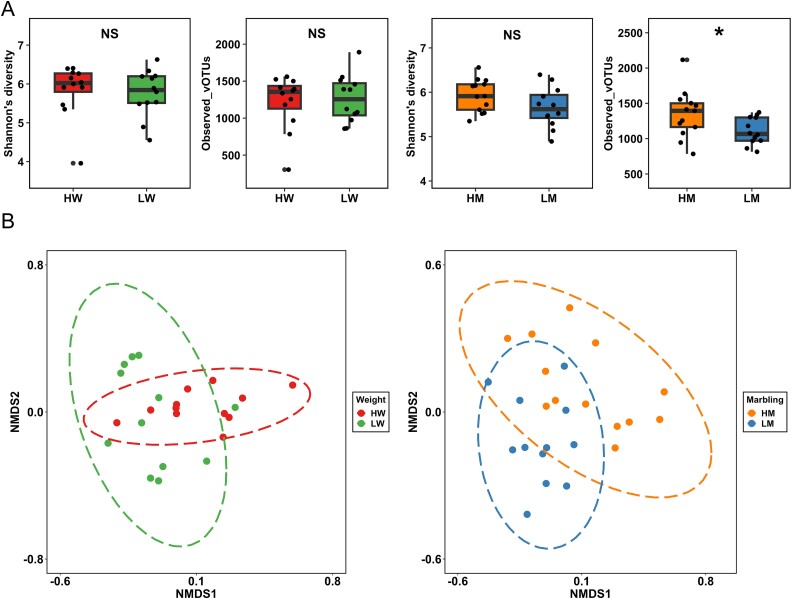
Characteristics of the rumen virome of the Japanese Black cattle. (A) Boxplot of α-diversity indexes based on vOTUs comparing HW vs. LW and HM and LM. (B) Principal coordinate analysis plot of *β*-diversity based on vOTUs showing differences between HW vs. LW and HM and LM (*P* < .01). An asterisk (*) indicates statistical significance at *P* < .05. “NS” stands for “not significant” (*P* > .05).

The composition and functions of rumen prokaryotes have been linked to carcass traits [3–5]. For instance, marbling has been positively correlated with the alpha diversity of the rumen microbiome [4], and specific bacterial taxa, such as Verrucomicrobia and *Olsenella*, have been associated with marbling in beef cattle [5]. Additionally, the abundance of rumen prokaryotes involved in carbon and methane metabolisms has been shown to be higher in cattle with HW compared to those with LW [3]. Here, this study determined the relationship between rumen viruses and carcass traits, particularly marbling and CW, in Japanese Black cattle. In terms of alpha diversity, the observed vOTUs and VCs were significantly higher in the HM group than in the LM group (*P* < .05; Fig. 5A; Supplementary Fig. 1). However, no significant differences in alpha diversity were observed between the HW and LW groups (Fig. 5A; Supplementary Fig. 1). These findings differ from those of a previous study that reported an association between the alpha diversity of the prokaryotic community and CW, but not marbling, in the same animals analyzed in this study [3]. One possible explanation for this inconsistency is that most vOTUs in the reference database used for mapping were incomplete genomes. This may have introduced biases into the α-diversity analysis and affected the observed results.

**Figure 6:**
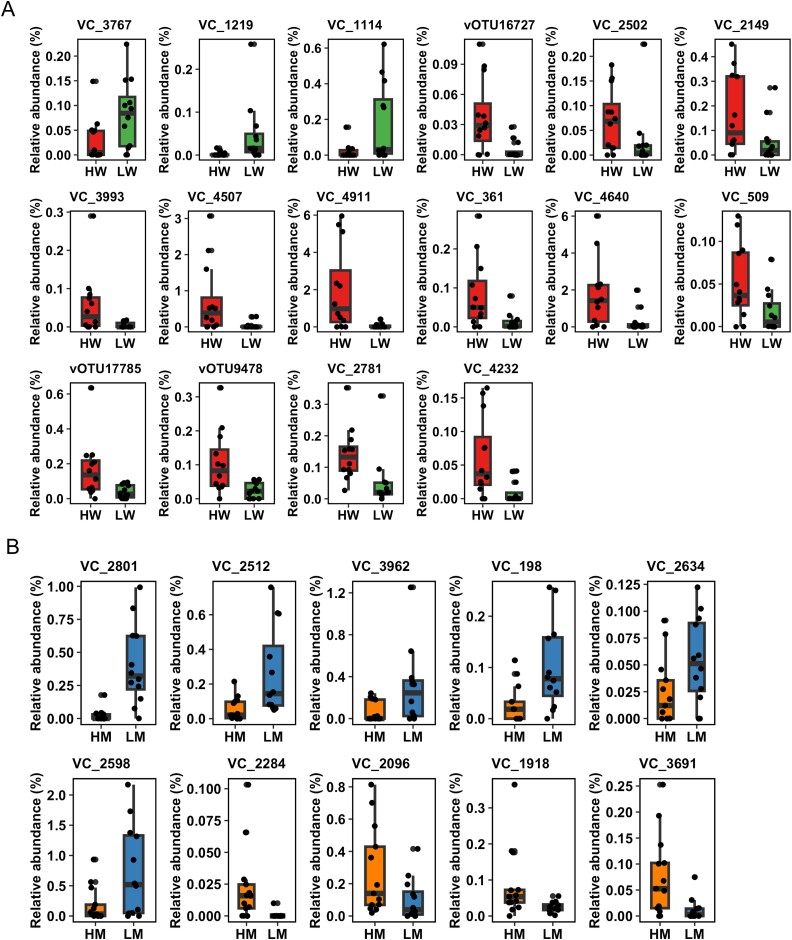
Box plot of the relative abundance of VCs in the rumen of the Japanese Black cattle. Comparisons between (A) HW vs. LW and (B) HM and LM. VCs with a significant Pearson correlation (*P* < .05) and a significant difference in Mann–Whitney’s U test with Benjamini–Hochberg correction (q < 0.1) are presented.

Bray–Curtis dissimilarity was calculated at the vOTU and VC levels to evaluate differences in rumen virome between HW vs. LW and HM vs. LM. NMDS plots using Bray–Curtis dissimilarities indicated a clear separation of rumen virome between HW vs. LW and HM vs. LM (Fig. 5B; Supplementary Fig. 2). Additionally, the PERMANOVA test revealed significant differences in rumen virome between HW vs. LW and HM vs. LM at both the vOTU and VC levels (*P* < .01). A previous study demonstrated that the prokaryote community, based on ASVs, exhibited a clear separation between HW vs. LW and HM vs. LM in the same samples used in this study [3], suggesting that the rumen virome changes considering the prokaryote community, potentially influencing CW and marbling in Japanese Black cattle, either directly or indirectly. No significant difference in the percentage of temperate viruses was observed between HW vs. LW and HM vs. LM (Supplementary Fig. 3). Due to the filtering effect of VLP enrichment, many prophages may have been excluded, potentially leading to an underestimation of the proportion of temperate viruses.

Pearson correlations were first calculated to determine the correlation between carcass traits and core VCs present in >50% of the samples. A total of 26 core VCs were significantly positively correlated with CW, while seven core VCs were significantly negatively correlated (*P* < .05; Supplementary Fig. 4). Additionally, four core VCs were positively correlated with BMS numbers, whereas 11 core VCs were negatively correlated (*P* < .05; Supplementary Fig. 5).

To further validate these correlations, the relative abundance of core VCs having evident correlations with CW and marbling was compared between the HW vs. LW and HM vs. LM groups. The relative abundance of 13 VCs was higher in the HW group than that in the LW group, whereas three VCs were more abundant in the LW group than that in the HW group (q < 0.1; Fig. 6A). Similarly, four VCs had higher relative abundance in the HM group than that in LM, whereas six VCs were more abundant in the LM group compared with that in the HM group (q < 0.1; Fig. 6B). These findings suggest that the abundance of specific viral genera is associated with carcass traits in beef cattle. Notably, VC_2781 and vOTU17785, predicted to associate with *Methanobrevibacter*, were more abundant in the HW group compared with that in the LW group. A previous study using the same rumen samples revealed that cattle with higher CWs had a higher abundance of methane-producing archaea, assigned to *Methanobrevibacter* [3]. Viruses can influence the rumen microbiome in a top–down or bottom–up manner [14, 62]. The top–down manner refers to viruses controlling host abundance by lysing them, while the bottom–up manner refers to viruses mediating or augmenting host metabolisms during infection. In this study, both host (*Methanobrevibacter*) and related VC abundances were simultaneously increased in the rumen of HW cattle. This suggests that VCs might augment methane metabolism in a bottom–up manner, facilitating the removal of H₂ and maintaining a low H₂ concentration in the rumen. The inhibition of methanogenesis can lead to H₂ accumulation, which inhibits the re-oxidation of microbial NADH and thereby impairs rumen fermentation [63, 64]. From this perspective, facilitating H₂ consumption in the rumen may improve fermentation efficiency, potentially leading to increased productivity and improved CW. However, no AMGs related to methane metabolism were found in the VCs in the present study. Additionally, the impact of H₂ accumulation on productivity remains unclear. Recent study suggests that the inhibition of methanogenesis and the accumulation of H₂ in the rumen do not appear to impair digestion and fermentation [65]. Other study demonstrated that the negative effects by inhibiting methanogenesis and the accumulation of H_2_ were not observed consistently in vivo [66]. Therefore, the reason why higher abundances of *Methanobrevibacter* and their viruses are associated with improved carcass traits remains unclear in this study. Further validation is needed to investigate the importance of VCs.

In addition, several VCs associated with bacteria involved in carbohydrate degradation in the rumen were more abundant in the HW group than in the LW group (q < 0.1; Fig. 6A). Among these, VC2149 and vOTU9478 were linked to *Prevotella*, while VC361 was associated with *CAG-791*. *Prevotella* is well-known for its role in carbohydrate degradation in the rumen [19–21]. Furthermore, *CAG-791* contains genes encoding GH5 (cellulase) and GH13 (amylases), which are involved in carbohydrate metabolism in the rumen [67]. A previous study also reported higher abundances of *Prevotella* in HW compared to LW [3], which may correspond to the increased abundance of viruses associated with *Prevotella*. These findings suggest that rumen viruses may influence carbohydrate metabolism, contributing to the observed increases in CW. However, no AMGs related to GHs were identified in vOTUs within these VCs, leaving their potential role in enhancing host carbohydrate metabolism unclear and requiring further investigation.

Regarding the interaction between viruses and marbling in beef cattle, the relative abundances of two VCs, VC2512 and VC263, predicted to associate with *Prevotella* were higher in the LM group than in the HM group (q < 0.1; Fig. 6B). This result is consistent with a previous study showing higher *Prevotella* abundance in the LM group than in the HM group [3]. On the other hand, VC3691, which is predicted to interact with *Treponema_D/_F*, had higher abundance in the HM group compared to the LM group (q < 0.1; Fig. 6B). *Treponema* has been associated with pectin degradation [68] and has been identified as a key contributor to lipid metabolism in the rumen of high-marbled beef [5]. Additionally, previous studies have shown higher abundances of *Treponema* in the rumen of Japanese Black cattle compared to crossbred cattle (Japanese Black sires × Holstein dams) [2]. Considering that Japanese Black cattle are capable of depositing large amounts of intramuscular fat [69] and generally exhibit higher marbling scores than crossbred cattle [70], *Treponema* may play an important role in marbling production. These findings suggest that the VCs interacting with *Treponema* may influence its abundance and functions, particularly in lipid metabolism, contributing to high marbling in beef cattle. Unfortunately, most of the hosts for other VCs associated with CW and marbling traits remain unidentified. Predicting the hosts of these VCs is critical for further elucidating the interactions between rumen viruses and carcass traits.

There are several limitations to this study. First, there may be limitations in accurately identifying the viral diversity, as the mapping rate was less than 50%. Additionally, a substantial number of viruses, particularly prophages, were filtered out during the purification process because VLP sequencing was used to investigate the rumen virome. Second, the core vOTUs and VCs identified in this study may not be core in other datasets because the rumen virome is highly independent [15]. Additionally, the study is missing data on several factors such as dietary feeds, age, and sex of the animals as previously mentioned [3], and cannot exclude the possibility that other factors affect the rumen virome. Due to these limitations, it is possible that the biological impact of the associated VCs on carcass traits could not be correctly identified. To address this, deep sequencing of both bulk and VLP samples is needed to capture viral diversity more accurately and the validation of our findings with additional datasets is necessary to confirm their robustness.

## Conclusions

In conclusion, this study identified the potential of rumen DNA viruses associated with CW and marbling in beef cattle using VLP sequencing of the rumen of Japanese Black cattle. Notably, VCs associated with *Methanobrevibacter* were linked to higher CW. Additionally, the study reconstructed 22 942 vOTUs of medium-quality or higher, referred to as JBRV, significantly expanding the publicly available catalog of rumen viral genomes. Many vOTUs within JBRV were found to interact with carbohydrate-degrading bacteria and contained AMGs involved in carbohydrate degradation, suggesting that rumen viruses may directly or indirectly influence carbon metabolism in the rumen. This database is valuable for future studies characterizing rumen DNA viruses and investigating their effects on the prokaryote community and host productivity.

## Supplementary Material

Supplementary_Table_1_ycaf021

Supplementary_Table_2_ycaf021

Supplementary_Table_3_ycaf021

Supplementary_Table_4_ycaf021

Supplementary_Figure_1-5_ycaf021

## Data Availability

Raw sequence data were deposited in DDBJ database (accession number PRJDB18783). The JBRV database can be accessed through FigShare (https://doi.org/10.6084/m9.figshare.26973358.v1).
