# Supplementary material for: Rumen DNA virome in beef cattle reveals an unexplored diverse community with potential links to carcass traits
Source: ISME Commun. 2025 Feb 5;5(1):ycaf021. doi: 10.1093/ismeco/ycaf021 (PMC11879238; doi:10.1093/ismeco/ycaf021)
Supplement: Supplementary_Figure_1-5_ycaf021 [file supplementary_figure_1-5_ycaf021.pdf]

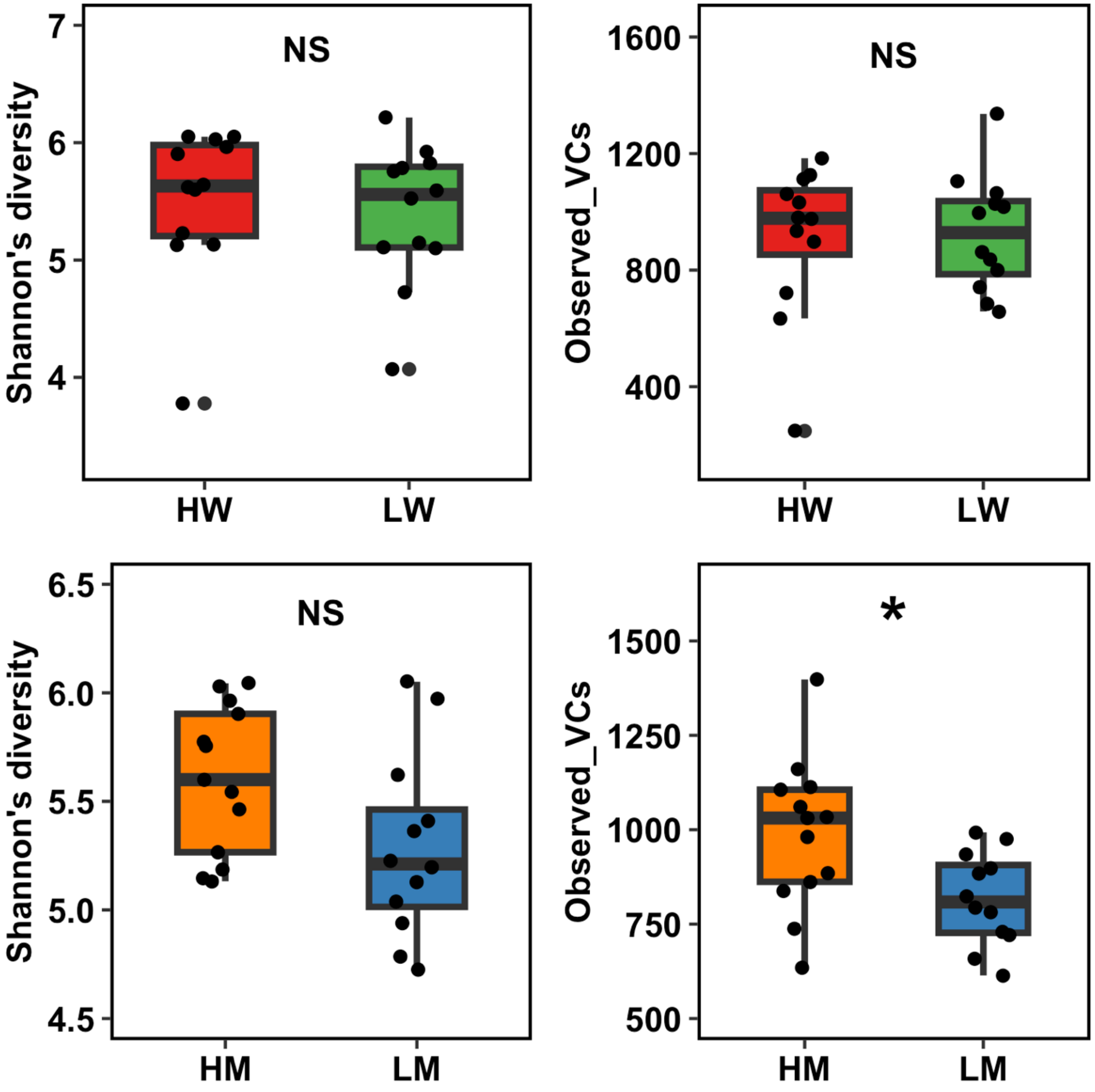


**Supplementary Fig. 1 Boxplot of α-diversity indexes based on viral clusters comparing HW vs. LW and HM vs. LM.** HM, high carcass weight, LW, low carcass weight.

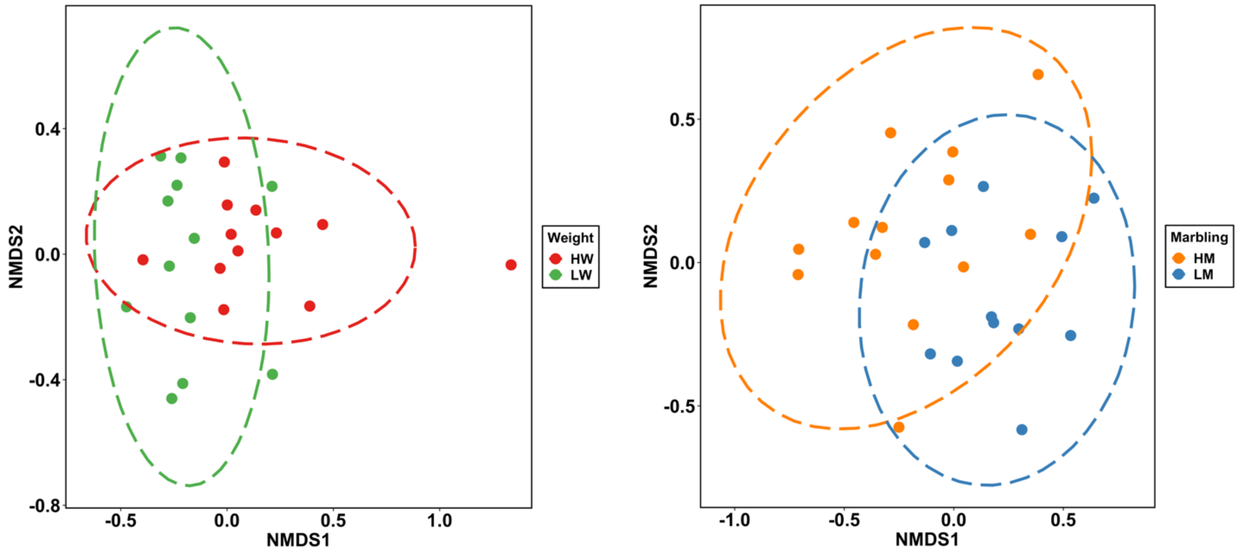


**Supplementary Fig. 2 Principal coordinate analysis plot of *β*-diversity based on viral clusters indicating differences between HW vs. LW and HM vs. LM.** HW, high carcass weight, LW, low carcass weight.

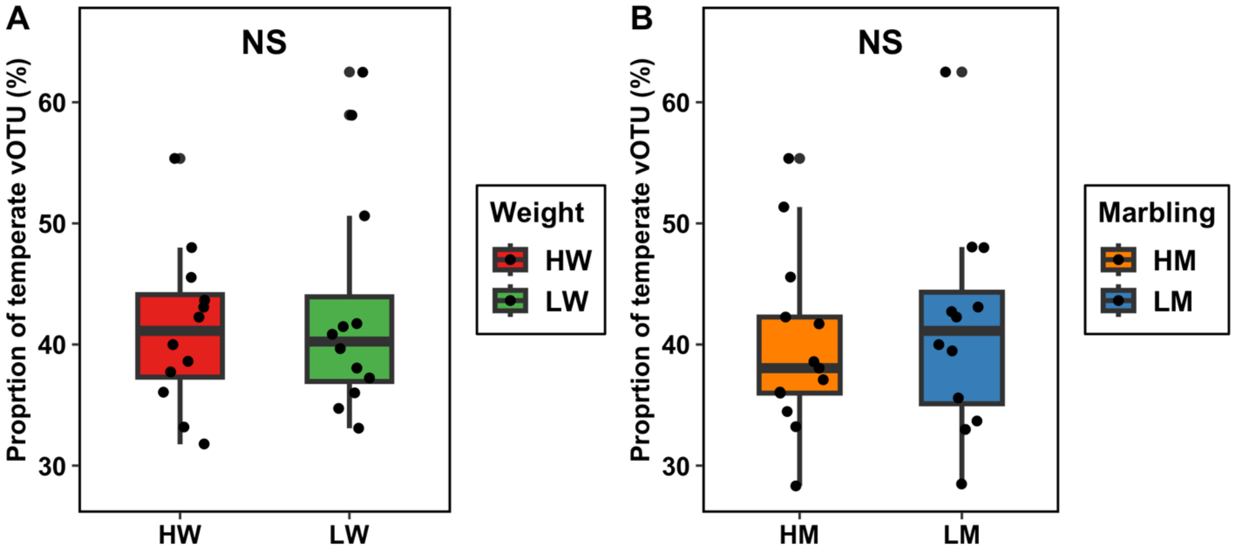


**Supplementary Fig. 3 Boxplot of the percentage of temperate viral operational taxonomic units comparing HW vs. LW and HM vs. LM.** "NS" stands for "not significant" ($p > 0.05$). HW, high carcass weight, LW, low carcass weight.

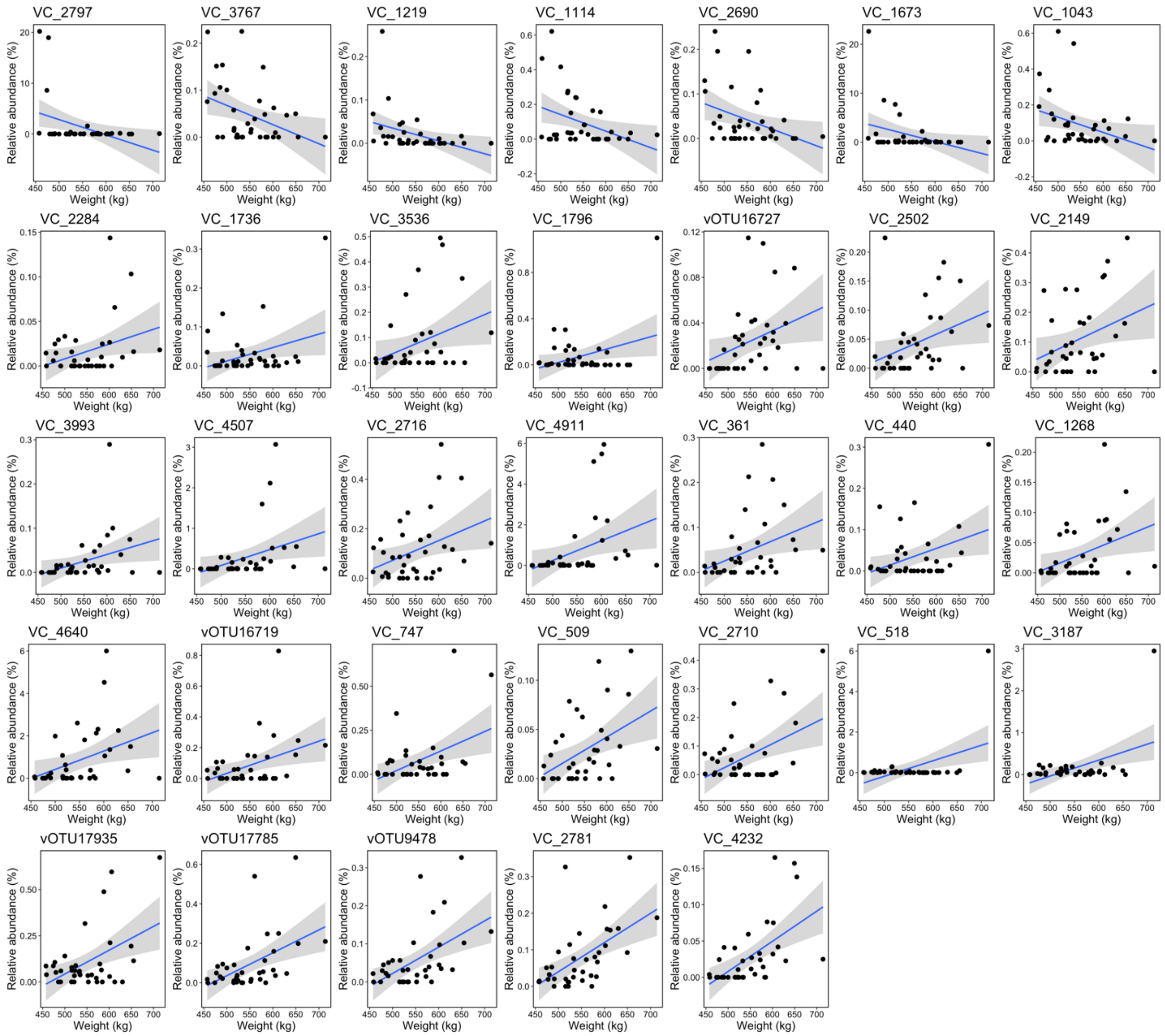


**Supplementary Fig. 4 Correlation between the relative abundance of core viral clusters (VCs) and carcass weight.** VCs with a significant Pearson correlation are presented ($p < 0.05$).

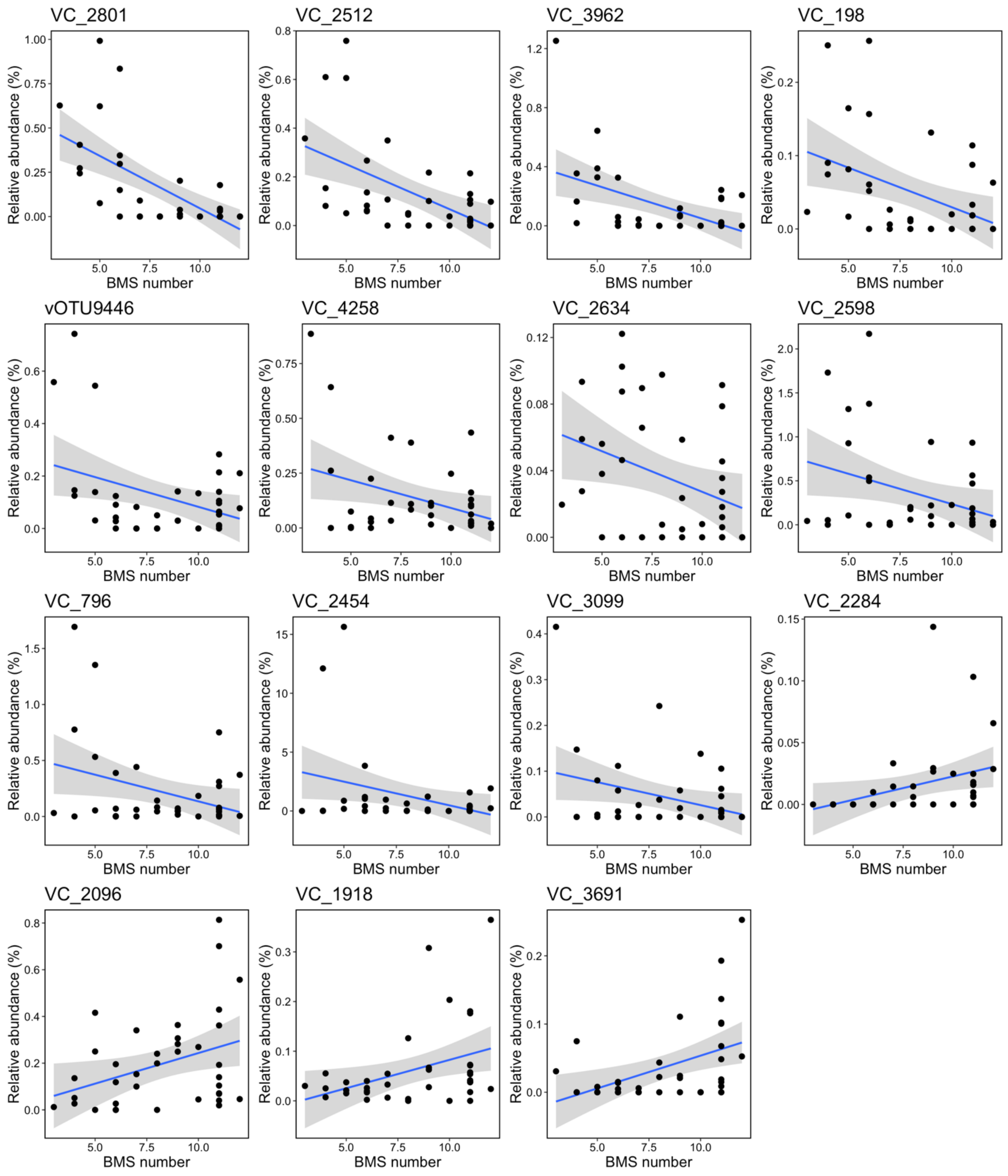


**Supplementary Fig. 5 Correlation between the relative abundance of core viral clusters (VCs) and marbling.** VCs with a significant Pearson correlation are presented ($p < 0.05$).
